# Sexual learning among East African adolescents in the context of generalized HIV epidemics: A systematic qualitative meta-synthesis

**DOI:** 10.1371/journal.pone.0173225

**Published:** 2017-03-09

**Authors:** Amelia S. Knopf, Kim R. McNealy, Halima Al-Khattab, Lisa Carter-Harris, Ukamaka Marian Oruche, Violet Naanyu, Claire Burke Draucker

**Affiliations:** 1 School of Nursing, Indiana University, Indianapolis, Indiana, United States of America; 2 Moi University School of Medicine, Eldoret, Rift Valley Province, Kenya; Vanderbilt University, UNITED STATES

## Abstract

**Background:**

AIDS-related illness is the leading cause of mortality for adolescents in sub-Saharan Africa. Together, Kenya, Tanzania, and Uganda account for 21% of HIV-infected adolescents in sub-Saharan Africa. The United Nations framework for addressing the epidemic among adolescents calls for comprehensive sexual and reproductive health education. These HIV prevention efforts could be informed by a synthesis of existing research about the formal and informal sexual education of adolescents in countries experiencing generalized epidemics. The purpose of this study was to describe the process of sexual learning among East African adolescents living in the context of generalized HIV epidemics.

**Methods:**

Qualitative metasynthesis, a systematic procedure for integrating the results of multiple qualitative studies addressing a similar phenomenon, was used. Thirty-two research reports met study inclusion criteria. The reports were assessed in a four-step analytic process: appraisal, classification of findings, synthesis of findings, and construction of a framework depicting the process of sexual learning in this population.

**Results:**

The framework includes three phases of sexual learning: 1) being primed for sex, 2) making sense of sex, and 3) having sexual experiences. Adolescents were primed for sex through gender norms, cultural practices, and economic structures as well as through conversations and formal instruction. They made sense of sex by acquiring information about sexual intercourse, reproduction and pregnancy, sexually transmitted infections, and relationships and by developing a variety of beliefs and attitudes about these topics. Some adolescents described having sexual experiences that met wants or needs, but many experienced sex that was coerced or violent. Whether sex was wanted, coerced, or violent, adolescents experienced worry about sexually transmitted infections or premarital pregnancy.

**Conclusions:**

The three phases of sexual learning interact to shape adolescents’ sexual lives and their risk for HIV infection. This framework will contribute to the development of sexual education programs that address HIV risk within the broader context of sexual learning.

## Introduction

Approximately 78% of adolescents living with HIV reside in sub-Saharan Africa, and AIDS is the leading cause of death in the region.[1, 2] The United Nations aims to end the AIDS epidemic by 2030. Ending the epidemic will require intense focus on key populations at high risk of acquiring HIV. Adolescents aged 10–19 accounted for 11% of incident HIV infections in 2013.[1] The Joint United Nations Programme on HIV/AIDS (UNAIDS) developed a strategic framework[1] for curbing new infections among adolescents. The framework has four underlying foci: 1) adolescent leadership, mobilization, and engagement; 2) human rights and equity; 3) sexual and reproductive health and education; 4) improved data to drive planning and results. The framework can guide efforts for HIV prevention, testing, and treatment among adolescent populations at highest risk of exposure to HIV, particularly in sub-Saharan Africa.

Three countries of interest, Kenya, The United Republic of Tanzania, and Uganda, all located in East Africa, account for 21% of the HIV infections among 10–19 year-olds living in sub-Saharan Africa.[2] In all three countries, the HIV epidemic is generalized, meaning that at least 1–5% of the general population is infected with HIV. The estimated HIV prevalence among 15–49 year-olds is 5.9% in Kenya, 4.7% in Tanzania, and 7.1% in Uganda. [2] In countries with 5% or higher HIV prevalence, no sexually active person can be considered “low risk.”[3]

Coitarche (or first sexual intercourse) is one of many transitions that typically occur in the second decade of life. Thus, for adolescents living in generalized HIV epidemic settings, adolescence marks a period of increased risk. Mitigating that risk requires a multi-pronged approach, such as the one proposed by UNAIDS. One of its core aims is to promote sexual and reproductive health and education. Adolescents’ knowledge and beliefs about HIV have been measured in all three countries through the Demographic and Health Surveys–AIDS Indicator Surveys (AIS). AIS conducted between 2011 and 2014 found that the youngest respondents (15–19 year-olds) were least likely to have comprehensive knowledge about HIV. Fewer than 40% of 15–19 year-olds in Uganda[4] and Tanzania[5] and 52% in Kenya[6] had comprehensive HIV knowledge.

Although we have a reasonable quantitative picture of Kenyan, Tanzanian, and Ugandan adolescents’ basic HIV knowledge, we know less about how they acquired it. Likewise, little is known about how these adolescents learn about sex and how sexual learning influences HIV risk. A number of researchers have conducted qualitative studies of adolescent sexual behaviors and HIV risk in eastern sub-Saharan African countries, however, their findings have not been synthesized to construct a picture of the broader context of adolescent sexual learning. The purpose of this qualitative metasynthesis was to characterize the process of sexual learning, as described by adolescents in three countries experiencing a generalized HIV epidemic—Kenya, Tanzania, and Uganda.

## Methods

Qualitative metasynthesis is a systematic procedure for evaluating and integrating the findings of multiple qualitative studies of a similar phenomenon.[7] This procedure was used in the current study to summarize knowledge generated by qualitative studies of adolescent sexual development in the three countries of interest in an effort to draw empirically based conclusions that are directly relevant to sexual health interventions and HIV prevention. A team comprised of four nurse researchers, two doctoral students in nursing, and one social scientist conducted this study. We used metasynthesis procedures developed by Sandelowski and Barroso.[7]

### Retrieving research reports

Reports were eligible for inclusion in this study if they (a) were published in English between 1/1/2003 and 31/12/2014; (b) used qualitative methodology; (c) addressed topics related to puberty, sex, reproduction, and/or intimate or dating relationships; and (d) included adolescents in Kenya, Tanzania, or Uganda.

English language was chosen because it is the official language shared by the three countries of interest as well as the shared language of all members of the research team. We restricted the search to reports published between 2003 and 2015 because we were most interested in understanding sexual learning in the current era of widely available testing and treatment for HIV. We chose 2003 as the starting point because The U.S. President’s Emergency Plan for AIDS Relief (PEPFAR) was initiated in that year. Through PEPFAR, the United States and its global partners have contributed more than $57 billion toward the prevention and treatment of HIV/AIDS.[8] The majority of PEPFAR funds have been spent in sub-Saharan Africa. The program is largely responsible for averting an estimated three million new infections and has provided treatment for nearly 10 million people living with HIV/AIDS.[9] Thus, 2003 serves as a natural dividing line between the early years of the African epidemic and the current period of intensive treatment and prevention efforts.

Sandelowski & Barroso recommend developing an initial set of search criteria, exploring the reports retrieved, and modifying as needed. We defined qualitative methodology broadly to include any empirical research with human subjects during which narratives that captured participants’ experiences were solicited and analyzed for patterns or themes. We focused on research conducted in Kenya, Tanzania, and Uganda because we were particularly interested in efforts to educate adolescents in East African countries with high burden of HIV among adolescents and adults. We included not only reports that addressed sexual activity but also reports that explored issues related to puberty, gender, reproduction, and intimate or dating relationships. We began our search using the World Health Organization’s definition of adolescence as the second decade of life, ages 10 to 20, inclusive, but later modified it for reasons explained in the results section, below.

Reports were excluded if they (a) included participants outside our age range of interest and findings attributable to these participants were indistinguishable from findings attributable to our designated population, (b) included participants from countries other than Kenya, Tanzania, and Uganda and findings attributable to those participants could not be distinguished from the findings attributable to participants in the countries of interest, or (c) were part of mixed methods studies whose quantitative findings could not be separated from qualitative findings.

We searched 15 electronic databases (Academic Search Premier, Anthropology Plus, CINAHL Plus, Education Source, ERIC, Family & Society Studies Worldwide, Family Studies Abstracts, MasterFile, MEDLINE, PsycArticles, PsycINFO, PubMed, Race Relations Abstracts, SocIndex, Social Work Abstracts) with search terms determined by the research team to be consistent with our inclusion criteria. The search terms for PubMed were: Sex* OR Reproduct* AND Adolescen* OR Teen* OR Youth* AND Kenya OR Tanzania OR Uganda AND Case study OR constant comparison analysis OR content analysis OR conversation analysis OR descriptive study OR discourse OR discourse analysis OR ethnograph* OR exploratory OR field observation OR field study OR focus group OR grounded theory OR hermeneutic OR interview OR narrative OR naturalistic OR participant observation OR phenomenology OR qualitative study OR qualitative research OR semiotic* OR thematic analysis. We limited our search to databases of interdisciplinary, peer-reviewed research reports to keep the search manageable and replicable. Two members of the research team independently reviewed the titles, abstracts, and full-text articles to determine which reports met all study criteria.

### Appraising research reports

We used a 14-item reading guide[7] to appraise multiple aspects of each of the qualitative reports. Sandelowski notes that despite decades of effort to devise criteria and methods for evaluating the quality of qualitative research studies, there is no census on the criteria or whether they should be applied at all. Efforts to codify quality in qualitative research risk losing scientifically relevant findings for reasons that do not render them unimportant. For example, if sample size is a criterion by which quality is assessed, one risks losing important findings in multiple small studies that were judged to be of low-quality for small sample rather than inappropriate methods. Thus, Sandelowski’s approach to appraising research reports focuses on differences in kind of qualitative findings, rather than quality of research studies.

Aspects of appraisal include the theoretical orientation of research, sample size and composition, efforts to maximize validity, and the logic and form of research findings. We used this guide to appraise articles that met inclusion criteria and constructed tables that displayed information from each report for each item on the reading guide. Two research team members independently evaluated each report and recorded their notes in tabulated format in Microsoft Word™. The team met weekly to discuss members’ appraisals of the reports so that all team members were familiar with the content of all the studies included in the analyses.

### Classifying study findings

Sandelowski and Barroso[7] propose a typology to classify the findings of reports based on the extent of the transformation of the raw data. Identification of the types of findings to be merged inform the approach to synthesis. These types, in ascending order of data transformation, include (1) no findings (i.e., presentation of uninterrupted data), (2) topical surveys (i.e., lists and inventories of topics), (3) thematic surveys (i.e., latent pattern of themes discerned from data), (4) conceptual/thematic description (i.e., concepts or themes developed in situ), and (5) interpretive explanation (i.e., fully integrated explanations of phenomenon). Two researchers evaluated each report and determined the level of data transformation. Where they disagreed, a third researcher helped reach consensus. Final classifications were entered in a cross-case comparison table to aid in the integration of the findings.

### Extracting, editing, and abstracting the study findings

The final set of reports was divided among members of the research team to extract the findings relevant to adolescent sexual learning. The findings were entered into a spreadsheet in which each line represented one research finding from a single report. Each line was then edited to form a stand-alone sentence that would make sense to a naïve reader. Two authors reviewed all edited statements and compared them to the original reports, making edits as necessary. We looked for topical similarities in these statements and further abstracted them until we arrived at a set of statements that concisely summarized all the findings.

### Determining the effect sizes of the abstracted statements

Sandelowski and Barroso[7] recommend the calculation of “manifest frequency effect sizes” for each abstracted statement to reflect the relative frequency with which the finding captured by the statement appeared in the sample of reports. A manifest frequency effect size is determined by taking the number of reports containing each finding (minus reports with the same finding from duplicate or overlapping samples) and dividing it by the total number of reports (minus those with common or overlapping samples).

### Taxonomic analysis

The final step of the metasynthesis was a taxonomic analysis. Whereas the effect sizes demonstrate the range of the magnitude of the findings, a taxonomic analysis demonstrates their conceptual range. Through a review of the edited and abstracted statements, with a return to the full-text reports when necessary, abstracted statements that shared a semantic relationship were grouped into categories. This grouping occurred through discussion and consensus within the research team. The categories were further grouped into conceptual domains representing processes that contributed to sexual learning among adolescents in Kenya, Tanzania, and Uganda.

## Results

**Fig 1:**
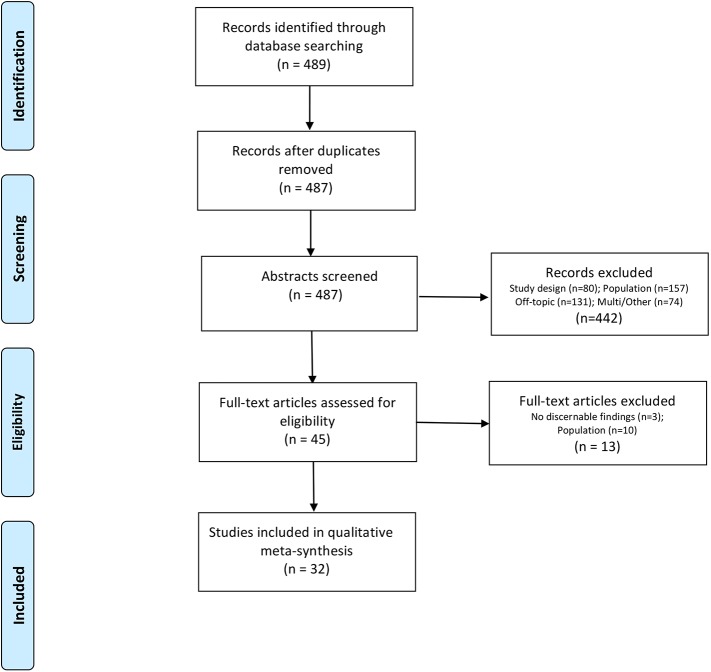
PRISMA diagram.

The search process is summarized in the PRISMA diagram, Fig 1 (S1 Table). The initial literature search yielded 487 unique reports. Two team members evaluated each abstract for inclusion in the study. These researchers modified the age range for inclusion for two reasons. First, they noted that few studies included adolescents as young as 10 years of age. Secondly, the few studies that did include younger adolescents also included children or adults outside the age range of interest, but did not label findings by age. Thus, it was difficult to extract findings only from study participants in the adolescent age range. Therefore, the final criteria for age were modified to ages 13–20, inclusive. Forty-five reports met inclusion criteria for full-text appraisal by members of the research team.

During the appraisal process, an additional 13 reports were rejected as we determined there were no discernible qualitative findings (n = 3) or we could not distinguish findings attributable to our designated population from findings attributable to other groups included in the study samples (n = 10). Thus, thirty-two reports were retained for synthesis.

The final 32 research reports[10–41] reflected a wide range of disciplines and methodological perspectives. The majority of primary authors was affiliated with academic institutions (n = 24) and represented the following disciplines: public health (n = 17), sociology (n = 3), education (n = 2), medicine (n = 2), demography (n = 1), geography (n = 1), development (n = 1), and gender studies (n = 1). Fourteen authors identified that the following theoretical frameworks guided their research: Feminist Theory (n = 3), Social Learning Theory (n = 2), Social Ecological Model (n = 1), Discourse Theory (n = 1), Theory of Gender Empowerment (n = 1), Theory of the Social Construction of Gender (n = 1), Transition to Adulthood Framework (n = 1), Resilience Theory (n = 1), Social Constructivism (n = 1), Jaccard’s Framework of Parent-Adolescent Communication (n = 1) and Baumrind’s Parenting Styles Framework (n = 1). The research methodologies described in the reports included qualitative description (n = 15), phenomenology (n = 3), ethnography (n = 2), and grounded theory (n = 2). In addition, participatory (n = 2), narrative/discursive (n = 1), and social constructivist (n = 1) approaches were identified.

There were 29 unique samples; 4 reports were generated from a single sample. Sample sizes ranged from 7 to 735 participants, and 10 studies had sample sizes larger than 100. The reports represented a total of 3,594 participants. Three reports did not specify the total number of male and female participants in the sample. Among the rest, 59% of the sample was female (*n* = 1,956) and 41% was male (*n* = 1,368). Research took place in rural areas (*n* = 16), cities (*n* = 9), or multiple sites that included both urban and rural residents (*n* = 4). Twelve samples were from Kenya, six were from Tanzania, and 11 from Uganda.

The extraction and editing of findings resulted in 711 unique statements, which were grouped as described in the methods section, above, into a set of 63 abstracted statements (S2 Table). The frequency effect sizes ranged between .03 and .38. The 14 statements with frequency effect sizes > 0.20 are presented in Table 1. A frequency effect size of .20 means the finding appeared in about one-fifth of reports with unique samples (n = 29).

**Table 1:** Abstracted findings with effect sizes >0.20.

Abstracted Finding	Effect Size
Although some adolescents have considerable and accurate knowledge about HIV transmission, others have no information or hold a variety of inaccurate beliefs that cause them to underestimate risk of transmission.[11],[15],[23–25],[28–30],[34], [36,37]	38%
Though some adolescents have accurate knowledge about condoms and are willing to use them, others hold a variety of beliefs and attitudes that are at odds with condom use.[11–14],[16–17],[24],[28],[29],[33],[34]	38%
Adolescents experience anxiety about premarital pregnancy, which can have dire consequences for girls as they often are shamed, blamed, and rejected by their families; forced into early marriage; and/or required to leave school/quit their education.[14],[22],[25],[27],[28],[32],[34–37],[39]	38%
Adolescents, especially girls, experience a wide variety of types of sexual coercion and violence perpetrated by peers, intimate partners, and familiar adults. [10],[13–15],[17],[19],[27–28],[31],[32],[36],[38]	38%
Some girls engage in transactional sex freely for love, to meet basic needs, or to gain access to luxury items, whereas others are pressured by family members (for their financial gain), and still others are coerced by older men.[10],[14],[17],[19],[24–25],[32],[34–35],[37–38]	38%
Adolescents learn about HIV from a variety of sources and would like to know more about HIV infection and other STIs, including the symptoms of infection, how HIV/STIs are transmitted, how to safely interact with those who are infected, and how to reduce their own risk of infection.[11],[13],[15–17],[28–30],[34],[36]	34%
Communication between adolescents and parents about issues of sex, pregnancy, and HIV/STIs is facilitated if parents are receptive and reassuring, but can be impeded if parents are unaware of the adolescent’s sexual learning needs, drink too much alcohol, or experience depleting life burdens.[11],[13],[18],[21–23],[30],[36]	28%
Girls may be forced into early marriages for the family’s financial benefit, but then face further financial hardship and have limited decision-making power, especially in regard to sex and reproduction.[14],[17],[20],[26],[35],[37],[38]	24%
Girls receive mixed messages about expectations for their sexual behavior–they are expected to avoid romantic relationships, or to be hesitant of sex, and also agreeable to satisfying males’ sexual desires, whereas boys receive a consistent message that they are expected to be overbearing, persistent, and even forceful in their pursuit of sex.[17],[22],[24],[27],[31–32],[34],[38]	24%
Men are expected to provide material or financial support for women so transactional sex between boys and girls can be either part of a normal, healthy love relationships or can be coercive.[14],[15],[24–25],[31],[38]	21%
Adolescents are curious and uncertain about puberty, adolescence, and sexuality but are not well informed and maintain inaccurate beliefs about the relationships among these factors.[13],[24],[28],[34–36]	21%
Although adolescents desire communication about or information on topics related to sexual learning, they often experience barriers that include lack of “space” in which to deal with or express their sexual feelings and difficulty communicating with adults because of fear of social and physical consequences.[11],[13],[18],[22],[28],[36]	21%
Adolescents are reluctant to approach parents for advice about sexual relationships or menstruation, even when they would like parental advice.[11],[18],[21–23],[30]	21%
Adolescent girls described a range of factors that influenced their sexual debut, and many felt unprepared for the social and emotional consequences of sexual debut.[14],[20],[27],[31],[34],[38]	21%

**Fig 2:**
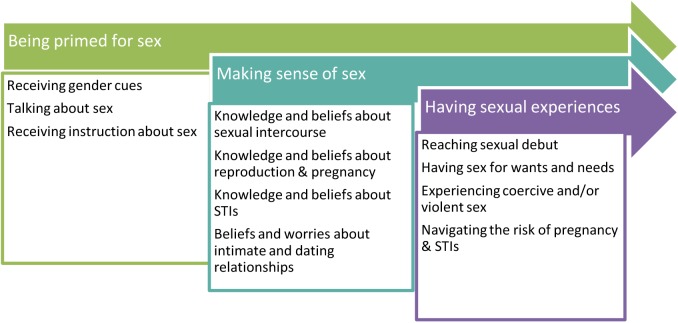
The process of sexual learning in three east African countries with high HIV prevalence.

Through a review of the 63 abstracted statements, with a return to the full-text reports when necessary, those that shared a semantic relationship were grouped into 11 categories. These 11 categories were further abstracted into three conceptual domains. We labeled these three domains as *being primed for sex*, *making sense of sex*, and *having sexual experiences*. The domains could be placed along a continuum that represents a conceptual rendering of sexual learning as a developmental process across childhood and adolescence. The three domains are described below and depicted in Fig 2.

### Being primed for sex

We labeled the first domain *being primed for sex* because the adolescents’ sexual learning began with messages they received about puberty, sex, reproduction, and/or intimate or dating relationships throughout childhood, often long before they engaged in sexual activities. These messages were received in three ways. Many messages about gender were rooted in the adolescents’ everyday experience or observation of domestic life and social practices in their families and communities. We refer to this process as “receiving gender cues” because the messages were not necessarily articulated but were implicit in the cultural contexts in which the adolescents grew up. Other messages about sexuality came more explicitly from those close to the adolescents. We referred to this process as “talking about sex” because the messages occurred in the context of interpersonal interactions. The third way in which adolescents were primed for sex occurred through teachings about sexuality in schools and religious institutions. We call this process “receiving instruction about sex” to reflect the more formal nature of these messages.

#### Receiving gender cues

The participants learned from an early age that young men and women are afforded different opportunities in their communities and have different expectations for behavior. The gendered nature of their social world thus provides the context of their sexual learning. Young women, for example, are provided fewer educational opportunities. For many, school is not valued or emphasized, and young women are more likely to drop out than are their male peers. Young women have considerable domestic responsibilities, which limit their exposure to people and ideas outside the home. Young women also have few opportunities to earn money and are actively discouraged from working. They are sometimes pressured into early marriage by parents, guardians, or siblings to reduce the financial burden of the young women’s care and so their families can profit from a wedding dowry. Young women who are orphaned or living in female-headed households are particularly vulnerable to early marriage and pregnancy, which effectively ends their educational trajectory. Young men, on the other hand, have greater access to education and are less likely to have their education interrupted. They have greater freedom to socialize outside the home and thus have larger social networks that provide knowledge and access to economic opportunities.

Adolescents receive cues about expectations for sexual and romantic relationships, and these also differ for young women and men. Young women feel pressure to remain abstinent or risk humiliation, rejection, and even violence from their parents and community, and yet they often face harassment and hostility if they refuse sexual advances. On the other hand, young men are expected to initiate relationships and pursue multiple partners. They often insist on sex from young women, at times in an overbearing manner. Some young men are encouraged by peers to “pester” young women for sex in public settings such as along the roadside or at village functions such as weddings and funerals. Young men hear older men’s stories of sexual exploits, which include tales of multiple partnerships, sexual pleasure, and tricking female partners out of condom use.

Cues about gender and sex also come from sources outside of the community. Pornography is available to adolescents through a variety of venues, including film, print media, and online. Young women view pornography as a negative influence, whereas young men find pornographic films instructive. In addition to viewing films, some young men access pornography through the Internet. They also use the Internet to interact with peers on social media, find dates, and learn more about “being modern.”[40] Young women use the Internet to socialize with friends away from the restrictions imposed at home and school, but they are fearful of disclosing their online activities because of the perception that Internet use is less acceptable for young women than for young men.

#### Talking about sex

Adolescents are also primed for sex through interactions they have with those close to them, including family members and peers. Many adolescents would like to have direct conversations with parents and grandparents about a wide range of topics related to sexual learning. They are curious about puberty and the emotional and physical changes they experience as they mature and want to know more about the context and meaning of sex and how to navigate sexual and romantic relationships. They would like reassurance that what they are experiencing in puberty is normal, and they desire advice about their transition into adulthood. Young women would like more information from their mothers, aunts, and grandmothers or from other older women in the community about menstruation and sex. Some young women wished for the traditional growing-up rituals that gave prior generations “deep” guidance about relationships with men, although other young women associate these rituals with female genital cutting and do not desire their return. Many adolescents therefore feel parents and other adults have an important role to play in the adolescents’ sexual learning, especially in helping them prevent HIV among youth.

Despite their desire for more discussions about topics related to sexual learning, many adolescents encounter a number of barriers to initiating such conversations. These adolescents feel uncomfortable breeching long-standing cultural traditions that prohibit frank parent-child discussions about sex. They also worry their parents will take the adolescents’ questions about sex as indicative of sexual activity and humiliate or punish them. Adolescents are especially reluctant to approach parents who are harsh, abuse alcohol, work long hours, or are abusive. Some adolescents perceive their parents as too consumed with just trying to survive to talk about sex.

Parents who do engage in discussions about sex with their adolescents often speak about it indirectly. Adolescents are told to “use good behavior”[25] to avoid HIV, “stay away from boys”[22] or “be careful” to avoid “diseases.”[15] Some parents threaten violence if they discover their adolescents are engaged in sexual activities. The fear-based messages from parents are a significant obstacle to open discussions about not only sex but also puberty and menstruation. Some young women, for example, are afraid to tell their mothers they began menstruating because they worry the menstrual blood will be misconstrued as a sign of having “bad behavior.”[36]

Despite these difficulties, some adolescents communicate with their parents about sex, especially if the adolescents view their parents as warm, available, and trustworthy. Some parents go against traditional taboos and talk about a broad array of topics including changes of puberty, care of the body, and HIV. Young women whose mothers discuss menstruation with them value their mothers’ advice and consider it trustworthy and memorable. Some parents initiate these conversations after observing other adolescents’ behavior, which they use as a touchstone for sharing their views on sexual intercourse and the importance of being aware of HIV and other STI. Some parents are health care providers and speak frankly about sex, condom use, and STI prevention.

Adolescents also talk with peers in and out of school about their dating or intimate relationships, their sexual experiences, and concerns about sexually transmitted infections, especially HIV. Peers often share stories about what they have “heard” about sexual matters. For example, they commonly discuss people they knew who died of AIDS and speculate on how their “reputation” might have contributed to their infection. Some peers encourage each other to protect their sexual health. In one instance, for example, young male street vendors encouraged other vendors to focus on their work and bring money back home to their parents rather than engaging in sex with young women.[15] Some school-going young women bring back knowledge they acquired at school to their peers who were forced to quit school. Young women who have access to AIDS education may share their knowledge by answering questions posed by girls who do not. Talking with peers about sex, however, can also have harmful consequences. Adolescents may misinform each other about the risks of pregnancy and STIs, pressure each other to have sex, and damage each other’s reputations by spreading gossip about their peers’ sexual activities.

#### Receiving instruction about sex

Adolescents receive some formal instruction about puberty, sex, reproduction, and intimate or dating relationships in school. However, this instruction is often incomplete and inadequate from the adolescents’ perspective. Teachers primarily focus on HIV prevention and often emphasize abstinence. In addition, some teachers provide misinformation. In one instance, Tanzanian primary school teachers told young women that their mothers might die if they discussed menarche together.[36] Students often hear a variety of inaccurate statements about condoms, including that the condoms may be contaminated with HIV, are effective in preventing pregnancy but not HIV, and are for use only by adults.

Outside of the school setting adolescents receive formal instruction related to their sexual learning in a few other venues. For instance, some young men receive formal instruction in sexual health through circumcision rites. For initiates, circumcision marks a transition to “manhood,” and many experience significant peer pressure to “prove their manhood” by having sex with young women. In some communities, parents and community leaders organize church-based circumcision rites that provide broad sexual education aimed to combat attitudes that pose a health risk to young men. However, these circumcision retreats are expensive and available only to young men from families with financial resources.

Some adolescents receive instruction from health care workers and see them as important and credible sources of information. Other adolescents do not receive services from healthcare workers or attend clinics because the adolescents fear other patients or the healthcare providers themselves will breach confidentiality. Finally, some adolescents receive instruction in HIV prevention from government-sponsored educational entertainment programs, including plays, pamphlets, and videos.

### Making sense of sex

As a result of being primed for sex in their families and communities, adolescents acquire knowledge and develop a variety of beliefs and attitudes about sex. Knowledge and beliefs regarding sex determine how adolescents understand sexual intercourse, reproduction and pregnancy, sexually transmitted infections, and intimate and dating relationships. Therefore, we call this second domain *making sense of sex*.

#### Knowledge and beliefs about sexual intercourse

Adolescents have mixed opinions about sex. Some think having sex is a natural part of maturing and associate sex with pleasure, but many view premarital sex as wrong or immoral. Many adolescents believe young men have sex for pleasure whereas young women have sex to meet material needs. Young men see sex as a way to occupy or entertain themselves or to make progress in a relationship, whereas young women see sex as preparation for marriage and a safeguard against future temptations. Prior to having sexual intercourse, adolescents often do not understand sexual mechanics and want to know more about how and why people have sexual intercourse.

#### Knowledge and beliefs about reproduction and pregnancy

Some adolescents do not understand the physical changes they experience as they reach puberty, and others are confused about the relationship between puberty and sex. For example, some adolescents think that having sex soon after reaching puberty will protect procreative potential, prevent painful menstruation, and facilitate a young woman’s physical maturation to improve birth outcomes later in life. Young women are largely unprepared for menarche. Some are shocked, embarrassed, ashamed, or confused when they begin menstruating and attempt to hide menstruation because they worry about being accused of sexual activity. They do not understand their menstrual cycles and wish their teachers, mothers, or older female relatives would provide instruction about managing menses before or at menarche.

Few adolescents understand how a woman becomes pregnant. Some young women correlate virility with age and think that having sex with younger males will protect them from pregnancy. Others think that one cannot become pregnant at coitarche or during coitus of short duration. Many are unable to identify the times in a woman’s cycle that pregnancy can be avoided, have misinformation about contraceptives, and are unsure about how to prevent pregnancy.

Adolescents have strong opinions about abortion, and would prefer to carry an unplanned pregnancy to term rather than terminate it.[39] Some view pregnancy as a punishment for the sin of premarital sex and believe that carrying a fetus to term will atone for this sin. Others believe abortions pose risks to their psychological and physical health but prefer those risks to the social and economic consequences of a premarital pregnancy. Some adolescents claim they would terminate an unplanned pregnancy to avoid social stigma and to remain in school.

#### Knowledge and beliefs about sexually transmitted infections

Knowledge about sexually transmitted infections varies widely. Some adolescents have accurate knowledge about HIV and its prevention. They know that HIV can be spread through blood transfusions, exposure to contaminated sharps, exchange of bodily fluids during sex, and from an infected mother to her child. They know condoms can prevent both HIV and pregnancy, and they have some general awareness that they are at risk of becoming infected if they have sex without condoms. Other adolescents have inaccurate knowledge about HIV. They think HIV can be acquired by routine daily activities, such as contact with the bedding of infected persons or from sharing their food or water. Some do not attribute their risk to their own or their partner’s sexual behavior but instead to sharing sharp objects like needles or razors or through getting a haircut at a barbershop. Some adolescents are convinced they can determine whether a person has HIV by his/her appearance, or they believe they can eliminate risk of exposure by having sex with very young partners.

Adolescents maintain a variety of beliefs that are at odds with condom use for STI prevention. Some conduct their own non-sexual experiments with condoms (e.g., filling them with water, rubbing them with hot peppers to see if the skin underneath still burns) that lead them to conclude condoms are not effective at preventing disease transmission. In one instance, adolescents in rural Kenya espoused the belief that condom use is emasculating and real men do not use condoms.[24] Adolescents are privy to a public discourse that casts doubts on the safety of condoms, especially for prevention of HIV, and may hear that condoms are contaminated with HIV during manufacturing, dangerous to young women who may be killed or injured by their use, and too large for young men to use.

Many adolescents believe condoms are for use in new relationships, when trust between partners has not yet been established. They associate condoms with a lack of faithfulness or mistrust in the relationship. Young women rarely receive instruction about condom use, and some are told condoms are only for “bad girls” and “prostitutes.” They are therefore unlikely to insist on continued condom use within the context of a stable partnership, even if that is what they would prefer. Some believe that oral contraceptives are preferable to condoms in the context of stable relationships but may not be aware that oral contraceptives will not prevent HIV.

#### Beliefs, attitudes, and worries about intimate and dating relationships

Young women and young men are confused by new emotions they experience during puberty and are curious about changes they observe in social dynamics between the sexes. They sense that there is some code of behavior for interactions between young men and women and may become frustrated and angry because they do not understand it. They are worried about being isolated and lonely if they do not have a boyfriend or girlfriend, but feel uncertain about how to behave around the opposite sex and how to become intimate with a partner. Young women are concerned and frustrated by the double standards for men and women and feel pressured to keep their relationships with young men a secret to avoid parental anger or rejection. They also worry about whether it is right to have a boyfriend and feel unsure about how to respond to advances from their male peers.

Adolescents have clear ideas about the ideal partner. Young women believe the ideal partner is a young man with a good reputation who is not known for promiscuity or womanizing. Young women often want a steady partner who is slightly older, earns an income, and is not infected with HIV. Young men believe the ideal partner is well-behaved, beautiful, and interested in bearing their children. Although some young men are interested in a long-term stable partner, they believe casual partnerships are less work than on-going ones. Some adolescents are aware of risks associated with multiple and concurrent partnerships, and some espouse that faithfulness to one partner can be facilitated through love, respect, communication, and sexual satisfaction. Some think fear of disease may also promote fidelity but are aware that fidelity can be compromised by money and transactional sex. Though many adolescents value faithfulness, most believe it is difficult to achieve and impossible to guarantee.

### Having sexual experiences

We labeled the third domain *having sexual experiences* to capture the broad range of sexual activities experienced by adolescents. Starting with their sexual debut, their sexual experiences can be motivated by their wants and needs or can result from violence. Regardless of the circumstances of their sexual experience, pregnancy and STI transmission are basic concerns once adolescents begin to experience sex.

#### Sexual debut

Adolescents’ sexual debuts are meaningful and memorable experiences that result in a range of emotions. Young women especially reflect on their debut and often consider it as something that happened to them rather than something they chose. Some feel conflicted about the timing and circumstances of their first sexual experience and question if they really wanted it. Many believe they were not prepared for the emotional and physical consequences of sex. Young men often view their sexual debuts as an entry into manhood. Some feel pressured into sexual debuts because it is what is expected of them.

#### Sex for “wants” or “needs”

Adolescents’ motivations for having sex vary according to their life circumstances. Many engage in sexual activities based on their emotional and physical desires (their “wants”) or to obtain tangible things they lack (their “needs”). Although motivations for being sexually active are complex, adolescents typically report having sex primarily because they desire it or primarily because the sex will provide for their basic needs.

The sexual “wants” of adolescents differ according to gender. Young men often enter into sexual relationships to experience pleasure, pass time, entertain themselves, or prove their “manliness” to peers. Young women, on the other hand, often enter into sexual relationships out of curiosity, under pressure from peers, to fulfill sexual desire, or because they are “in love” or want to get married.

Other adolescents have sex as a form of economic exchange. Based on cultural expectations, young men routinely provide gifts or money to their sexual partners. Gifts vary in type and value but often include toiletries (soaps, lotions), snacks (chips, soda), schoolbooks, clothing, or cash. Some young women view this exchange as a normal part of a sexual relationship, whereas others, particularly young women who are orphans or who come from very poor households, believe they must have sex to secure food and necessities for their families or themselves. For example, sometimes older male family members, relatives, or other authority figures provide school fees in return for sex with the young women. Some young women specifically seek out older men for sexual partners because they typically have more money and can provide better gifts, whereas other young women feel trapped into sex with an older man because of family expectations or poverty.

#### Sexual violence

Many adolescents experience sex in the context of violence. We use the term violence broadly to include a wide variety of forced and abusive sexual practices. Young women in particular are often victims of verbal harassment, sexual coercion, physical aggression, unwanted sexual touching, and rape. These acts of violence are committed by strangers, acquaintances, peers, relatives, and partners, as discussed below.

Young women routinely experience verbal harassment in the form of name-calling, insults, ridicule, and persistent demands for sex in the course of their daily lives. Many must deal with harassment in their homes, on their way to school or work, at school, or at community events such as weddings or funerals. The young women feel embarrassed, degraded, humiliated, and powerless in response to such harassment.

Young women also routinely experience sexual coercion. Many are aggressively “pestered” for sex by peers. Some young women believe this is wrong and a “good” boyfriend is one who allows the woman to make choices about sex. Older men often threaten violence, punishment, abandonment, or the withdrawal of money or material goods if young women fail to comply with their sexual demands. Although sexual coercion is most commonly directed toward young women, young men can experience it as well. Some young men are pressured into sex by older women, and some young men are pressured to have sex with young women by peers and older adult men who challenge young men to prove they are “men.” In some instances, male peers threaten or use physical violence against young men who fail to engage in sex, as is expected of them.

Young women also may experience physical aggression from men who have targeted the young women for sex. For example, men may block young women’s paths to school and grope, shove, or push them if they do not respond to the men’s sexual comments or invitations. Young women also experience unwanted touching from their male peers at school. The young men may sit close to the young women in the classroom, fondle them, and then claim the young women are just pretending that they do not want to be touched.

Some young women experience forced sexual penetration or rape by strangers, family friends, peers, boyfriends, or spouses. Rapes occur in a variety of contexts. Some young women are raped while walking between households at night or when going to and from school. Others are raped in their own homes or in the homes of relatives, friends, or partners. Still others are raped at community gatherings, especially those that involve heavy drinking such as weddings and funerals. Adolescents’ understanding of what constitutes rape varies. Some consider it rape if a woman is forced to have sexual intercourse to which she did not consent, even if this occurs in the context of marriage or an established relationship. Most believe rape must include physical force. Many believe women cannot be raped by husbands or established partners since forced sex is an expected and acceptable part of male and female relationships.

Adolescents believe young women are harmed by sexual violence but acknowledge a number of cultural beliefs and attitudes about gender roles that support sexual violence. For example, in one instance young women claimed that it was their duty to unconditionally satisfy a male partner’s desires because men cannot withstand abstinence.[38] Thus, they expected coercion and forced sex within intimate partnerships and did not consider it rape. Adolescents believe a victim of sexual violence may be to blame for her own experience because of her clothing choices, her behavior (e.g., walking alone at night), or poor decision-making (e.g., accepting a ride from a stranger). Some young women think it is best to acquiesce to men’s demands to avoid painful sex, physical violence, and instability in romantic relationships.

Adolescents are often silent about the sexual violence they experience. Even if they wanted to report this violence to authorities, they believe they would be admonished for doing so. They are concerned about not being taken seriously, being blamed for their own abuse, being further victimized, or being shunned by their communities for being “spoiled.” They worry about bringing shame on their families if they report the crime, and most see more disadvantages than advantages to reporting. Some adolescents are reluctant to disclose an assault to a parent because they fear being blamed, and others are afraid to report assaults perpetrated by friends, relatives, or family friends. Adolescents are not sure what types of sexual violence they could report and worry about reporting crimes for which they have no proof. They lack confidence in their ability to articulate what has happened to them and believe they would not be believed or supported. Adolescents would like to know how and to whom to report sexual violence and what to do in response to abuse. They also want to know how they can avoid coercion and rape.

Young women experience a number of psychological consequences of sexual violence. They feel degraded and powerless and have difficulty concentrating in school. They may attend classes but find concentration is difficult following an assault. They feel ashamed and worried about being shunned by their families and communities if anyone were to discover they had been assaulted.

#### Risk of pregnancy and STIs

Regardless of the circumstances of adolescents’ sexual experiences, many struggle with the accompanying risk of pregnancy and STI transmission. The use of condoms, for example, often creates conflict within their sexual relationships. Some young women wish to carry condoms and insist their partners use them, but fear that asking a partner to use a condom will give the impression that they are promiscuous, unfaithful, or infected with HIV and thus their reputation in the community will be damaged. Young men, on the other hand, are seen as responsible if they carry or use condoms.

Some young women discuss the risk of pregnancy with their partners and may tentatively request they use a condom. Partners may agree to use condoms but then claim to have forgotten them, not wear them and hope their partners do not notice, or remove them during intercourse. Other young men may counter their partners’ request for condoms by demanding more frequent intercourse. Despite these barriers, some young women do assert themselves in their sexual relationships and demand condom use. In addition to condoms, some women also use oral contraceptives or practice periodic abstinence or withdrawal prior to ejaculation. Some young women use folk remedies, including herbs, to prevent pregnancy or STIs.

Adolescents who do become infected with an STI face barriers to diagnosis and treatment. They are reluctant to visit government-run health clinics because of privacy concerns and are worried that the provider would know their parents and report the visit. They are also concerned about seeing people from the community in the waiting areas and causing speculation about the reason for the visit. Few can afford the costs of screening and medications. Some adolescents try using traditional medicines or herbs to treat some STIs such as gonorrhea.

Young women who are not married and become pregnant often suffer dire consequences. They are blamed for the pregnancy, face social stigma, and have limited life choices. Many persons view premarital pregnancy as shameful, irresponsible, and immoral. Although laws exist to criminalize sex with women younger than age 18, the laws are not enforced and young men typically deny paternity to avoid conviction. When paternity is denied, young women are left to provide for themselves and their child(ren), which is difficult for them to do. Therefore, some young women who become pregnant are forced into early marriages in which their power to make decisions about sex and reproduction are constrained.

## Discussion

Our qualitative metasynthesis of 32 research reports reveals a process of sexual learning in adolescents living in three East African countries experiencing generalized HIV epidemics. Our findings indicate that adolescents’ sexual experiences are best understood in the context of life-long sexual learning that spans the period from receiving gender-based cues about sexuality as children to having sexual experiences in which they are confronted with negotiating prevention of pregnancy and STIs, especially HIV, in adolescence. The model presented in Fig 2, therefore, represents a complex interaction of factors that can render adolescents at risk for HIV transmission. These factors can include gendered cultural norms, misinformation about sexual risk from a variety of sources, scarcity of resources and practices of economic exchange for sex, prohibitions about open discussions of sexual concerns, and the prevalence of sexual violence.

The model suggests multiple pathways through which HIV transmission can occur in adolescence. Cultural norms about gender, for example, may require a young woman to be “pure” but privilege a young man’s sexual needs, resulting in her being shamed or punished if she acquiesces to demands for sex or, conversely, harassed, rejected, or subjected to violence if she refuses these demands. Other cultural norms about gender may encourage a young man to “prove” his manhood following circumcision, and because he believes condoms to be ineffective, contaminated, and dangerous to a young woman’s health, he does not consider their use, thereby increasing the risk of HIV transmission in all his sexual encounters.

The findings in this review resonate with those of several other studies. For example, a needs assessment conducted in Nyanza Province, Kenya, found that adolescents desired more communication with adults, especially parents, and that their lack of knowledge about sex and HIV contributed significantly to poor sexual health outcomes.[42] The needs assessment is consistent with our finding that many adolescents lack knowledge about sexual issues and wish to discuss their concerns with important adults in their lives (Table 1).

The reports included in our review indicated that some teachers overemphasize the risk of HIV posed by sharp objects, underemphasize sexual risk, and reinforce myths about condom use[11] and menstruation.[36] These findings are consistent with those of Gallant and Maticka-Tyndale,[43] who examined the effectiveness of 11 school-based HIV prevention programs in sub-Saharan Africa. Most of the programs were designed to increase knowledge about HIV/AIDS and to change attitudes toward people living with HIV/AIDS, though some also promoted abstinence, and, less frequently, condom use. Program content was strongly influenced by community norms and teachers’ perceptions of them. In their study, teachers resisted or refused to include content on condoms, even when students were sexually active.[43]

Findings that school-based education programs often did not provide adequate information was likely influenced by U.S. policies such as PEPFAR. In its first five years (2003–2008), one third of prevention funding was allocated to abstinence-only education programs that targeted adolescents. Although this requirement was later relaxed, between 2004 and 2013 PEPFAR provided $1.4 billion in sub-Saharan Africa for abstinence and faithfulness programming.[44] These programs had no significant effect on age of sexual debut, teenage pregnancy rates, or number of sexual partnerships in a 12-month period,[44] and they illustrate how U.S. policy affects or, in this case, does not affect global health outcomes.

The findings that adolescents have a variety of beliefs and attitudes that discourage condom use is consistent with findings from the AIS that indicate that in the countries of interest, condom use among adolescents is low. Roughly two thirds of adolescents in Kenya, Tanzania, and Uganda between the ages of 15 and 19 say they know where to purchase condoms. However, reports of condom use at last sexual activity remain low, especially for young women.[4–6]

Shifting to the most abstracted level of our results, the three domains of sexual learning, we find similarities between the sexual learning of Western adolescents and the east African adolescents represented here. For example, Fortenberry[45] describes Western adolescents’ sexual learning as an interaction between formal and informal sexual education and lived sexual experiences. Their formal sexual education is largely school-based, and much like that of the east African adolescents, its content varies but generally focuses more on the prevention of STI than the mechanics or subjective experience of sexual intercourse. Western adolescents’ informal sources of sexual education are similar to their east African counterparts: family members, peers, religious organizations, media, and observation of social interactions in their daily lives. In the West, as in east Africa, adolescents use their sexual knowledge to inform and interpret sexual experiences.[45]

In summary, at the most abstract level, the domains of sexual learning are similar across contexts, which has implications for HIV prevention. It suggests that the bidirectional exchange of research and program evaluation is important. The Parents Matter! program and Families Matter! programs are a useful exemplar. Parents Matter! was developed and tested in the U.S., where it significantly improved communication about sex, pregnancy, and HIV between parents and adolescents.[46] It was adapted for use in Nyanza Province, Kenya, and has since been implemented in eight sub-Saharan African countries where its impact is currently being evaluated.[47] This is just one example of the cultural adaptation of existing evidence-based HIV prevention curricula. It underscores the importance of cross-cultural exchange of ideas, and provides a template[47] for adapting successful interventions from one context to the other.

Our study has several limitations. First, our results are based on the findings of other researchers, which are necessarily limited by the questions they posed, the findings they presented, and the extent to which their findings were attributable to specific subgroups of participants. Thus, we may be missing key elements of the sexual learning process, or how it may differ for younger adolescents compared to older adolescents, or those who are married compared to those who are not. We noted few findings about the role of religious beliefs and religious institutions in shaping adolescents’ sexual development; only one report explored this in detail, leaving gaps in our understanding of the role of religion in east African context. We also noted a general lack of focus on positive influences on adolescents’ sexual learning. Finally, there were no findings about aspects of the adolescents’ sexual experiences that might have been satisfying or enjoyable.

We speculate that because the authors of the reports included in the review often aimed to address the problematic aspects of adolescent sexual activity, such as HIV transmission, they focused on contexts of risk rather than positive aspects of sexual learning. In addition, adolescent participants may not have felt comfortable acknowledging sexual pleasure to researchers or may simply have had no expectations of positive emotional or physiologic effects from their sexual experiences. Future research that provides ample opportunity for adolescents to share all aspects of their sexual learning could yield information to enrich sexual education programs by explicating protective as well as risk factors related to HIV transmission.

A second limitation of the reviewed reports is the lack of information about young people’s lived experience with HIV and other STIs, despite the high prevalence of the problem in the population studied here. It is possible the adolescent participants were unaware of their HIV status, because many of the studies were conducted just before or simultaneous to the time when anti-retroviral medications became available in the region and widespread testing began. Alternatively, adolescents with HIV may have been hesitant to expose themselves to the possibility of HIV-related stigma. In either case, more research is needed to understand the lives and challenges of adolescents who have acquired HIV, as described from their own perspectives.

Despite these limitations, the findings of these studies have practice and policy implications. Prevention programs in the region need to address how social context issues, such as gender norms, community practices, and economic conditions, influence sexual learning in adolescents and how this learning affects transmission of HIV. The multiple pathways to HIV transmission should be considered when prevention curricula are planned. In the schools, teachers need more training and community support for instructing young people about sexual risk reduction. HIV transmission is undoubtedly linked to the high prevalence of sexual violence, and both social problems might be addressed jointly in all prevention efforts.

## Conclusion

Young men and women in Kenya, Tanzania, and Uganda are maturing in the context of a generalized HIV epidemic in their communities. The framework of sexual learning that we propose is based on the narratives of a large number adolescents collected through a range of strategies including individual interviews, focus groups, role plays, and written responses to questions. Although the framework requires further testing, it provides a dynamic and contextualized description of sexual learning by adolescents in the region of interest and highlights some important factors that contribute to HIV transmission. Understanding sexual learning in increasingly nuanced ways by considering the voices of adolescents, including those affected by HIV, and others in their social world will contribute to the development of more effective prevention initiatives in the region.

## Supporting information

S1 Table PRISMA checklist. (DOCX)

S2 Table Abstracted statements (DOCX)

## References

[1] United Nations Joint Programme on HIV/AIDS. All In. 2015.

[2] Unicef. Seventh Stocktaking Report 2016: HIV/AIDS Statistical Tables. 2016.

[3] United Nations Joint Programme on HIV/AIDS. UNAIDS Prevention Toolkit 2016 [June 27, 2016]. Available from: http://hivpreventiontoolkit.unaids.org/.

[4] Uganda Ministry of Health and ICF International. Uganda AIDS Indicator Survey, 2011. Calverton, MD: 2012.

[5] Tanzania Commission for AIDS ZAC, National Bureau of Statistics, Office of Chief Government Statistician, and ICF International. Tanzania AIDS Indicator Survey, 2011–12. Calverton, MD: 2013.

[6] Kenya National Bureau of Statistics MoH, National AIDS Control Council, Kenya Medical Research Institute, National Council for Population Health and Development, and the DHS Program, ICF International. Kenya Demographic and Health Survey, 2014. Rockville, MD.

[7] Sandelowski MB J.. Handbook for Synthesizing Qualitative Research. New York, NY: Springer Publishing Company, Inc.; 2007.

[8] PEPFAR. PEPFAR funding fact sheet, 2016 2016 [June 28, 2016]. Available from: http://www.pepfar.gov/documents/organization/252516.pdf.

[9] Heaton LM, Bouey PD, Fu J, Stover J, Fowler TB, Lyerla R, et al Estimating the impact of the US President's Emergency Plan for AIDS Relief on HIV treatment and prevention programmes in Africa. Sexually transmitted infections. 2015;91(8):615–20. 10.1136/sextrans-2014-051991 26056389

[10] Abuya BA, Onsomu E.O., Moore D. & Sagwe J.. A phenomenological study of sexual harassment and violence among girls attending high schools in urban slums, Nairobi, Kenya. Journal of School Violence. 2012;11(4):323–44.

[11] Bastien S. Access, agency and ambiguity: communication about AIDS among young people in Northern Tanzania. Culture, health & sexuality. 2009;11(8):751–65. 10.1080/13691050903362632 20183551

[12] Baumgartner JN, Lugina H, Johnson L, Nyamhanga T. "Being faithful" in a sexual relationship: perceptions of Tanzanian adolescents in the context of HIV and pregnancy prevention. AIDS care. 2010;22(9):1153–8. 10.1080/09540121003615095 20824568

[13] Belita A, Kulane A., Ahlberg B.M. Adolescence and sexuality in the context of HIV and AIDS: Views and concerns of pupils in a rural primary school in Kenya. Child Health & Education: An Interdisciplinary Journal. 2011;3(2).

[14] Bell SA, Aggleton P. Economic vulnerability and young people's sexual relationships in rural Uganda. J Youth Stud. 2014;17(6):814–28.

[15] Birungi R, Nabembezi D, Kiwanuka J, Ybarra M, Bull S. Adolescents' perceptions of sexual coercion in Uganda. African journal of AIDS research: AJAR. 2011;10(4):487–94. 10.2989/16085906.2011.646664 25865380

[16] Chacko S, Kipp W, Laing L, Kabagambe G. Knowledge of and perceptions about sexually transmitted diseases and pregnancy: a qualitative study among adolescent students in Uganda. Journal of health, population, and nutrition. 2007;25(3):319–27. PubMed Central PMCID: PMC2754036. 18330065 PMC2754036

[17] Conn C. Young African women must have empowering and receptive social environments for HIV prevention. AIDS care. 2013;25(3):273–80. 10.1080/09540121.2012.712659 22908853

[18] Crichton J, Ibisomi L, Gyimah SO. Mother-daughter communication about sexual maturation, abstinence and unintended pregnancy: Experiences from an informal settlement in Nairobi, Kenya. J Adolescence. 2012;35(1):21–30. 10.1016/j.adolescence.2011.06.008 21783241

[19] Hayer MK. Perceptions of sexual coercion among young women in Uganda. Journal of health organization and management. 2010;24(5):498–504. 10.1108/14777261011070510 21033643

[20] Juma M, Askew I, Alaii J, Bartholomew LK, van den Borne B. Cultural practices and sexual risk behaviour among adolescent orphans and non-orphans: a qualitative study on perceptions from a community in Western Kenya. BMC public health. 2014;14:84 PubMed Central PMCID: PMC3912900. 10.1186/1471-2458-14-84 24467940 PMC3912900

[21] Kajula LJ, Sheon N, De Vries H, Kaaya SF, Aaro LE. Dynamics of parent-adolescent communication on sexual health and HIV/AIDS in Tanzania. AIDS and behavior. 2014;18 Suppl 1:S69–74. 24101099 10.1007/s10461-013-0634-6

[22] Kamau A, Bornemann R., Laaser U. Psychosocial influences on adolescent sexuality and identity in rural Kenya. Health Sociology Review. 2006;15(3):305–16.

[23] Lofgren J, Byamugisha J, Tillgren P, Rubenson B. The perspectives of in-school youths in Kampala, Uganda, on the role of parents in HIV prevention. African journal of AIDS research: AJAR. 2009;8(2):193–200. 10.2989/AJAR.2009.8.2.7.859 25875570

[24] Maticka-Tyndale E, Kyeremeh C. The Trouble With Condoms: Norms and Meanings of Sexuality and Condom Use Among School-Going Youth in Kenya. Int J Sex Health. 2010;22(4):234–47.

[25] Mmari K, Michaelis A, Kiro K. Risk and protective factors for HIV among orphans and non-orphans in Tanzania. Culture, health & sexuality. 2009;11(8):799–809. 10.1080/13691050902919085 19499394

[26] Mojola SA. Multiple transitions and HIV risk among orphaned Kenyan schoolgirls. Studies in family planning. 2011;42(1):29–40. PubMed Central PMCID: PMC3518408. 21500699 10.1111/j.1728-4465.2011.00262.x PMC3518408

[27] Muhanguzi FK. Gender and sexual vulnerability of young women in Africa: experiences of young girls in secondary schools in Uganda. Culture Health & Sexuality. 2011;13(6):713–25. 10.1080/13691058.2011.571290 21516536

[28] Njue C, Voeten H., Ahlberg B.M. Youth in a void: Sexuality, HIV/AIDS and communication in Kenyan public schools. Sex Education: Sexuality, Society and Learning. 2011;11(4):459–70.

[29] Njue C, Voeten HA, Remes P. Disco funerals: a risk situation for HIV infection among youth in Kisumu, Kenya. Aids. 2009;23(4):505–9. PubMed Central PMCID: PMC2675523. 10.1097/QAD.0b013e32832605d0 19165086 PMC2675523

[30] Nobelius AM, Kalina B., Pool R., Whitworth J., Chesters J., Power R.. Sexual and reproductive health information sources preferred by out-of-school adolescents in rural southwest Uganda. Sex Education 2010;10(1):91–107.

[31] Nobelius AM, Kalina B, Pool R, Whitworth J, Chesters J, Power R. "You still need to give her a token of appreciation": the meaning of the exchange of money in the sexual relationships of out-of-school adolescents in rural southwest Uganda. Journal of sex research. 2010;47(5):490–503. 10.1080/00224499.2010.494776 20628949

[32] Nobelius AM, Kalina B, Pool R, Whitworth J, Chesters J, Power R. Sexual partner types and related sexual health risk among out-of-school adolescents in rural south-west Uganda. AIDS care. 2011;23(2):252–9. 10.1080/09540121.2010.507736 21259139

[33] Nobelius AM, Kalina B, Pool R, Whitworth J, Chesters J, Power R. "The young ones are the condom generation": condom use amongst out-of-school adolescents in rural southwest Uganda. Journal of sex research. 2012;49(1):88–102. 10.1080/00224499.2011.568126 21516591

[34] Nzioka C. Unwanted pregnancy and sexually transmitted infection among young women in rural Kenya. Culture, health & sexuality. 2004;6(1):31–44. 10.1080/1369105031000106365 21972831

[35] Sekiwunga R, Whyte SR. Poor parenting: teenagers' views on adolescent pregnancies in eastern Uganda. African journal of reproductive health. 2009;13(4):113–27. 20690279

[36] Sommer M. Ideologies of sexuality, menstruation and risk: girls' experiences of puberty and schooling in northern Tanzania. Culture Health & Sexuality. 2009;11(4):383–98. 10.1080/13691050902722372 19326264

[37] Tolley EE, Kaaya S, Kaale A, Minja A, Bangapi D, Kalungura H, et al Comparing patterns of sexual risk among adolescent and young women in a mixed-method study in Tanzania: implications for adolescent participation in HIV prevention trials. Journal of the International AIDS Society. 2014;17(3 Suppl 2):19149. PubMed Central PMCID: PMC4163993. 25224611 10.7448/IAS.17.3.19149 PMC4163993

[38] Wagman J, Baumgartner JN, Waszak Geary C, Nakyanjo N, Ddaaki WG, Serwadda D, et al Experiences of sexual coercion among adolescent women: qualitative findings from Rakai district, Uganda. Journal of interpersonal violence. 2009;24(12):2073–95. 10.1177/0886260508327707 19109534

[39] Mitchell EM, Halpern CT, Kamathi EM, Owino S. Social scripts and stark realities: Kenyan adolescents' abortion discourse. Culture, health & sexuality. 2006;8(6):515–28. 10.1080/13691050600888400 17035171

[40] Pfeiffer C, Kleeb M, Mbelwa A, Ahorlu C. The use of social media among adolescents in Dar es Salaam and Mtwara, Tanzania. Reproductive health matters. 2014;22(43):178–86. 10.1016/S0968-8080(14)43756-X 24908469

[41] Puffer ES, Watt MH, Sikkema KJ, Ogwang-Odhiambo RA, Broverman SA. The protective role of religious coping in adolescents' responses to poverty and sexual decision-making in rural Kenya. Journal of research on adolescence: the official journal of the Society for Research on Adolescence. 2012;22(1):1–7. PubMed Central PMCID: PMC3325092. 22505794 10.1111/j.1532-7795.2011.00760.x PMC3325092

[42] Poulsen MN, Vandenhoudt H, Wyckoff SC, Obong'o CO, Ochura J, Njika G, et al Cultural adaptation of a U.S. evidence-based parenting intervention for rural Western Kenya: from parents matter! To families matter! AIDS education and prevention: official publication of the International Society for AIDS Education. 2010;22(4):273–85. 20707689 10.1521/aeap.2010.22.4.273

[43] Gallant M, Maticka-Tyndale E. School-based HIV prevention programmes for African youth. Social science & medicine. 2004;58(7):1337–51. 14759680 10.1016/S0277-9536(03)00331-9

[44] Lo NC, Lowe A, Bendavid E. Abstinence Funding Was Not Associated With Reductions In HIV Risk Behavior In Sub-Saharan Africa. Health affairs. 2016;35(5):856–63. 10.1377/hlthaff.2015.0828 27140992

[45] Fortenberry JD. Sexual Learning, Sexual Experience, and Healthy Adolescent Sex. New Dir Child Adoles. 2014;144:71–86. 10.1002/cad.20061 24962363

[46] Miller KS, Lin CY, Poulsen MN, Fasula A, Wyckoff SC, Forehand R, et al Enhancing HIV communication between parents and children: efficacy of the Parents Matter! Program. AIDS education and prevention: official publication of the International Society for AIDS Education. 2011;23(6):550–63. 22201238 10.1521/aeap.2011.23.6.550

[47] Miller KS, Lasswell SM, Riley DB, Poulsen MN. Families matter! Presexual risk prevention intervention. Am J Public Health. 2013;103(11):e16–20. PubMed Central PMCID: PMCPMC3828710. 10.2105/AJPH.2013.301417 24028229 PMC3828710

