# Supplementary material for: Sexual learning among East African adolescents in the context of generalized HIV epidemics: A systematic qualitative meta-synthesis
Source: PLoS One. 2017 Mar 9;12(3):e0173225. doi: 10.1371/journal.pone.0173225 (PMC5344379; doi:10.1371/journal.pone.0173225)
Supplement: S2 Table — (DOCX) [file pone.0173225.s002.docx]

|  | **Abstracted Finding** | **Effect Size** | **First Author of contributing reports** |
| --- | --- | --- | --- |
|  | Although some adolescents have considerable and accurate knowledge about HIV transmission, others have no information or hold a variety of inaccurate beliefs that cause them to underestimate risk of transmission. | 38% | Bastien; Birungi; Lofgren; Maticka-Tyndale; Mmari; Njue 2009; Njue 2011; Nobelius 2010a; Nzokia; Sommer; Tolley |
|  | Though some adolescent have accurate knowledge about condoms and are willing to use them, others hold a variety of beliefs and attitudes that are at odds with condom use. | 38% | Bastien; Baumgartner; Belita; Bell & Aggleton; Chacko; Conn; Maticka-Tyndale; Njue 2009; Njue 2011; Nobelius 2012; Nzokia |
|  | Adolescents experience anxiety about premarital pregnancy, which can have dire consequences for girls as they often are shamed, blamed and rejected by their families, forced into early marriage, and required to leave school/quit their education. | 38% | Bell & Aggelton; Kamau; Mitchell; Mmari; Muhanguzi; Njue 2011; Nobelius 2011; Nzokia; Sekiwunga; Sommer; Tolley |
|  | Adolescents, especially girls, experience a wide variety of types of sexual coercion and violence perpetrated by peers, intimate partners, and familiar adults. | 38% | Abuya; Belita; Bell & Aggleton; Birungi; Conn; Hayer; Muhanguzi; Nobelius 2010b; Nobelius 2011; Njue 2011; Sommer; Wagman |
|  | Some girls engage in transactional sex freely for love, to meet basic needs, or to gain access to luxury items, while others are pressured by family members (for their financial gain), and still others are coerced by older men. | 38% | Abuya; Bell & Aggelton; Conn; Hayer; Maticka-Tyndale; Mmari; Nobelius 2011; Nzokia; Sekiwunga; Tolley; Wagman |
|  | Adolescents learn about HIV from a variety of sources, and would like to know more about HIV infection and other STIs, including the symptoms of infection, how HIV/STIs are transmitted, how to safely interact with those who are infected, and how to reduce their own risk of infection. | 34% | Bastien; Belita; Birungi; Chacko; Conn; Njue 2009; Njue 2011; Nobelius 2010; Nzokia; Sommer |
|  | Communication between adolescents and parents about issues of sex, pregnancy, and HIV/STIs is facilitated if parents are receptive and reassuring, but can be impeded if parents are unaware about the adolescent’s sexual learning needs, drink too much alcohol, or experience depleting life burdens. | 28% | Bastien; Belita; Chrichton; Kajula; Kamau; Lofgren; Nobelius 2010a; Sommer |
|  | Girls may be forced into early marriages for the family’s financial benefit, but then face further financial hardship and have limited decision-making power, especially in regard to sex and reproduction. | 24% | Bell & Aggelton; Conn; Juma; Mojola; Sekiwunga; Tolley; Wagman |
|  | Girls receive mixed messages about expectations of their sexual behavior – they are expected to avoid romantic relationships, or to be hesitant of sex, and also agreeable to satisfying males’ sexual desires, whereas boys receive a consistent message that they are expected to be overbearing, persistent, and even forceful in their pursuit of sex. | 24% | Conn; Kamau; Maticka-Tyndale; Muhanguzi; Nobelius 2010b; Nobelius 2011; Nzokia; Wagman |
|  | Men are expected to provide material or financial support for women so transactional sex between boys and girls can be either part of a normal, healthy love relationships or can be coercive. | 21% | Bell & Aggelton; Birungi; Maticka-Tyndale; Mmari; Nobelius 2010b; Wagman |
|  | Adolescents are curious and uncertain about puberty, adolescence, and sexuality but are not well informed and maintain inaccurate beliefs about the relationships between these concepts. | 21% | Belita; Maticka-Tyndale; Njue 2011; Nzioka; Sekiwunga; Sommer |
|  | Although adolescents desire communication about or information on topics related to sexual learning, they often experience barriers that include lack to “space” in which to deal with or express their sexual feelings and difficulty communicating with adults due to fear of social and physical consequences. | 21% | Bastien; Belita; Chricton; Kamau; Njue 2011; Sommer |
|  | Adolescents are reluctant to approach parents for advice about sexual relationships or menstruation, even when they would like parental advice. | 21% | Bastien; Chrichton; Kajula; Kamau; Lofgren; Nobelius 2010a |
|  | Adolescent girls describe a range of factors that influenced their sexual debut, and many felt unprepared for the social and emotional consequences of sexual debut. | 21% | Bell & Aggleton; Juma; Muhanguzi; Nobelius 2010b; Nzioka; Wagman |
|  | Some adolescents are worried about contracting HIV, and therefore avoid activities they associate with risk of transmission such as sexual intercourse, being out alone at night and risking sexual violence, and caring for sick relatives. | .17 | Bastian; Mmari; Muhanguzi; Nobelius 2012; Nzioka |
|  | Adolescents believe their peers are more likely to engage in high risk sexual behaviors when they lack information about HIV, are curious about sex and see it as a game, are poor, feel unloved, or have parents who are too strict or too lenient. | .17 | Lofgren; Mmari; Mojola; Nzioka; Sekiwunga |
|  | Girls who engage in transactional sex gain small luxury items, essential goods, and cash but risk damage to their reputations, sexual violence, and the risks of unprotected sex | .17 | Abuya; Maticka-Tyndale; Mmari; Nobelius 2011; Wagman |
|  | The consequences of sexual coercion or violence for adolescents are many and include pregnancy and HIV/STIs, school problems, psychological distress, deterioration in quality of life, and lack of self-confidence. | .17 | Abuya; Conn; Hayer; Muhanguzi; Wagman |
|  | Adolescents are often silenced about the sexual violence and coercion they experience because reporting it or revealing it to others can result in rejection, stigmatization, retaliation, and violence. | .17 | Belita; Birungi; Muhanguzi; Nzioka; Wagman |
|  | Communication about or negotiation for condom use among partners can be impeded by social factors such as poverty as well as cultural beliefs and attitudes such as condoms are only for girls who are promiscuous. | .14 | Chacko; Conn; Nobelius 2012; Nzioka |
|  | Some boys make it difficult to use condoms by tampering with the condom, removing it during intercourse, or insisting on more frequent sexual intercourse when condoms are used. | .14 | Bell & Aggelton; Maticka-Tyndale; Nobelius 2012; Tolley |
|  | Girls use several strategies for avoiding unwanted transactional sex, including finding other means of supporting themselves (e.g. farming, selling vegetables), shaming men who attempt to lure them with gifts, praying for God to provide for them, and “detoothing” – frequently changing partners so one never has to “repay” for gifts with sex. | .14 | Bell & Aggelton; Mmari; Nobelius 2010a; Puffer |
|  | Adolescents have a number of worries about relationships including that that they will be lonely or rejected by peers if that do not have a partner, that they will not be able to form relationships, that they will be punished or have to quit school if they have a relationship, and they do not know how to act in relationships. | .14 | Belita; Kamau; Muhanguzi; Njue 2011 |
|  | Adolescents have limited knowledge about pregnancy and mixed opinions of contraception. | .14 | Belita; Chacko; Muhanguzi; Nzioka |
|  | Particular social and cultural practices in East Africa, such as disco funerals and widow inheritance, expose adolescents to risk of unprotected and coercive sex. | .14 | Juma; Njue 2009; Njue 2011; Sekiwunga |
|  | Adolescents have mixed opinions about premarital sex, and associate sex with both positive and negative outcomes. | .10 | Belita, Nzioka, Tolley |
|  | Condom use is generally more acceptable in casual or newly formed relationships, compared with long-term relationships. | .10 | Chacko; Nobelius 2011; Nobelius 2012; Tolley |
|  | Boys and girls see acceptance of a gift or money as a tacit agreement to have sex and therefore some expect boys to redress a “lack of payment” through sexual violence. | .10 | Abuya; Bell & Aggelton; Maticka-Tyndale |
|  | Girls engage in transactional sex with peers, familiar older men, and strangers, especially wealthy men whom they perceive as easier to manipulate into better gifts or longer financial support in the event of pregnancy. | .10 | Abuya; Maticka-Tyndale; Mojola |
|  | While some adolescents value communication related to sexual learning with older adults, including ssengas, grandparents, older siblings/cousins, and older adults in the community, the adolescents may avoid this communication as it will reveal they are sexually active or curious. | .10 | Belita; Mmari; Nobelius 2010a |
|  | Adolescents in east Africa value communication with peers about desired partners, negotiating relationships, and sexual health issues, although sometimes this communication involves unreliable information or pressure to form relationships. | .10 | Bastien; Nobelius 2010a; Sommer |
|  | When east African adolescents talk with their parents about sex, their messages are typically fear-based, threatening, and do not include practical advice about condoms or family planning. | .10 | Kajula; Nobelius 2010a; Sekiwunga |
|  | Some adolescent girls feel comfortable discussing contraception and pregnancy with their boyfriends, and use a variety of methods to prevent unintended pregnancy. | .10 | Chacko; Nzioka; Tolley |
|  | Most adolescents hold a negative opinion of abortion and would proceed with an unintended pregnancy, despite the potential social impacts on the mother’s life and risk of HIV transmission (if the mother were infected). | .10 | Chacko; Mitchell; Nzioka |
|  | Adolescents experience acts of violence in a variety of settings, including in homes, on school grounds, by the roadside en route to school or social events, and at large public gatherings. | .10 | Abuya; Birungi; Nobelius 2011 |
|  | A number of beliefs and attitudes related to gender roles support sexual violence and coercion. | .10 | Birungi; Conn; Wagman |
|  | Many east African adolescent girls find menarche and menstruation confusing and shameful, and would like to learn more about it. | .10 | chricton; Kamau; Sommer |
|  | Some adolescents receive messages in their communities that are inaccurate, such as condoms prevent pregnancy but not HIV. | .10 | Bastien; Nobelius 2010a; Sommer |
|  | Some adolescents believe HIV testing is important, even for stable, monogamous couples, but few know where to go for testing. | .07 | Baumgartner, Tolley |
|  | Boys may deny paternity to avoid conviction under defilement laws, which makes it very difficult for the girl to provide for her child(ren). | .07 | Bell & Aggelton; Sekiwunga |
|  | Adolescents experience uncertainty and mistrust of the opposite sex. | .07 | Belita; Sommer |
|  | Some adolescents receive information about sex, pregnancy, and HIV/STIs from teachers, churches, musawos (medics), and the media that is accurate, clear, presented in the local language, and privately disseminated. | .07 | Bastien; Nobelius 2010a |
|  | Adolescents who are not in school have restricted access to information about sexual development and HIV. | .07 | Bastien; Sekiwunga |
|  | While adolescent males view sexual relationships as a form of entertainment and as a means of progressing a relationship, females view sexual relationships as a means of preparing for marriage and managing sexual temptations. | .07 | Bell & Aggelton; Muhanguzi |
|  | Some girls believe that acquiescence is the best way to avoid aversive sexual experiences. | .07 | Conn, Wagman |
|  | Adolescents do not know how to respond to sexual violence and coercion, and would like skills that would help them avoid it and facilitate reporting it. | .07 | Belita; Birungi |
|  | Adolescents have heard HIV prevention messages about faithfulness, which have shaped their beliefs about concurrent and serial partnerships. | .03 | Baumgartner |
|  | Boys are thought to have sex for pleasure while girls are thought to have sex primarily to meet their material needs. | .03 | Nobelius 2010b |
|  | Boys who engage in transactional may find it challenging to meet girls’ ongoing demands for money. | .03 | Bell & Aggelton |
|  | Communication with older adults sometimes encourages sexual risk behaviors, especially for male adolescents who hear stories about older men’s sexual exploits. | .03 | Nobelius 2010a |
|  | Messages received in healthcare settings can be accurate but healthcare encounters may compromise the adolescents’ privacy. | .03 | Nobelius 2010a |
|  | Some boys receive formal instruction related to reducing sexual risk behaviors during circumcision rites organized by churches. | .03 | Kamau |
|  | Adolescents believe that while faithfulness is expected in marriage and possible in serial relationships, it is hard to ensure | .03 | Baumgartner |
|  | Adolescents believe faithfulness is enhanced by love, respect, and good communication between partners; marriage; sexual satisfaction; a harmonious household; and fear of disease, and is threatened by the transactional aspects of relationships and coercive sex. | .03 | Baumgartner |
|  | While girls wish for stable partners who have good behavior, a good reputation, are free of HIV/AIDs, appear healthy, are slightly older, and earn wages, boys want to find a well-behaved, beautiful steady partner who will bear their children. | .03 | Nobelius 2011 |
|  | Adolescents who are abstinent and those who are sexually active would like to know how to control sexual urges. | .03 | Belita |
|  | Adolescents use the internet to meet potential partners and to learn about sex and reproductive health. | .03 | Pfeiffer |
|  | Some girls consider forced sexual intercourse with an established intimate partner to be expected and do not believe it is rape or can be characterized as unwanted. | .03 | Wagman |
|  | East African adolescents believe rape is sex without consent, and usually involves physical violence. | .03 | Hayer |
|  | East African adolescent boys gain new social status at circumcision, and are often pressured to prove their manhood by having sex with girls soon afterward. | .03 | Kamau |
|  | Adolescents view sex as a natural part of adult life. | .03 | Maticka-Tyndale |
|  | Adolescents learn about sex through pornographic movies, but have mixed opinions about viewing these films. | .03 | Nobelius 2010a |
|  | Adolescents use prayer and religious coping to resist sexual temptations. | .03 | Puffer |

**Complete List of Reports**

Abuya BA, Onsomu, EO, Moore, D, Sagwe, J. A phenomenological study of sexual harassment and violence among girls attending high schools in urban slums, Nairobi, Kenya. *Journal of School Violence.* 2012;11(4): 323-344.

Bastien S. Access, agency and ambiguity: communication about AIDS among young people in Northern Tanzania. *Culture, Health & Sexuality.* Nov 2009;11(8): 751-765.

Baumgartner JN, Lugina H, Johnson L, Nyamhanga T. "Being faithful" in a sexual relationship: perceptions of Tanzanian adolescents in the context of HIV and pregnancy prevention. *AIDS Care.* Sep 2010; 22(9): 1153-1158.

Belita A, Kulane, A, & Ahlberg, BM. Adolescence and sexuality in the context of HIV and AIDS: Views and concerns of pupils in a rural primary school in Kenya. *Child Health & Education: An Interdisciplinary Journal.* 2011; 3(2).

Bell SA, Aggleton P. Economic vulnerability and young people's sexual relationships in rural Uganda. *Journal of Youth Studies.* 2014;17(6): 814-828.

Birungi R, Nabembezi D, Kiwanuka J, Ybarra M, Bull S. Adolescents' perceptions of sexual coercion in Uganda. *African Journal of AIDS Research : AJAR.* Dec 2011;10(4): 487-494.

Chacko S, Kipp W, Laing L, Kabagambe G. Knowledge of and perceptions about sexually transmitted diseases and pregnancy: a qualitative study among adolescent students in Uganda. *Journal of Health, Population, and Nutrition.* Sep 2007;25(3): 319-327.

Conn C. Young African women must have empowering and receptive social environments for HIV prevention. *AIDS Care.* 2013;25(3): 273-280.

Crichton J, Ibisomi L, Gyimah SO. Mother-daughter communication about sexual maturation, abstinence and unintended pregnancy: Experiences from an informal settlement in Nairobi, Kenya. *Journal of Adolescence.* Feb 2012;35(1): 21-30.

Hayer MK. Perceptions of sexual coercion among young women in Uganda. *Journal of Health Organization and Management.* 2010;24(5):498-504.

Juma M, Askew I, Alaii J, Bartholomew LK, van den Borne B. Cultural practices and sexual risk behaviour among adolescent orphans and non-orphans: a qualitative study on perceptions from a community in Western Kenya. *BMC Public Health.* 2014;14: 84.

Kajula LJ, Sheon N, De Vries H, Kaaya SF, Aaro LE. Dynamics of parent-adolescent communication on sexual health and HIV/AIDS in Tanzania. *AIDS and Behavior.* Jan 2014;18 Suppl 1: S69-74.

Kamau A, Bornemann, R, Laaser, U. Psychosocial influences on adolescent sexuality and identity in rural Kenya. *Health Sociology Review.* 2006;15(3): 305-316.

Lofgren J, Byamugisha J, Tillgren P, Rubenson B. The perspectives of in-school youths in Kampala, Uganda, on the role of parents in HIV prevention. *African Journal of AIDS Research : AJAR.* Jun 2009;8(2): 193-200.

Maticka-Tyndale E, Kyeremeh C. The Trouble With Condoms: Norms and Meanings of Sexuality and Condom Use Among School-Going Youth in Kenya. *International Journal of Sexual Health.* 2010;22(4): 234-247.

Mitchell EM, Halpern CT, Kamathi EM, Owino S. Social scripts and stark realities: Kenyan adolescents' abortion discourse. *Culture, Health & Sexuality.* Nov-Dec 2006;8(6): 515-528.

Mmari K, Michaelis A, Kiro K. Risk and protective factors for HIV among orphans and non-orphans in Tanzania. *Culture, Health & Sexuality.* Nov 2009;11(8): 799-809.

Mojola SA. Multiple transitions and HIV risk among orphaned Kenyan schoolgirls. *Studies in Family Planning.* Mar 2011;42(1): 29-40.

Muhanguzi FK. Gender and sexual vulnerability of young women in Africa: experiences of young girls in secondary schools in Uganda. *Culture Health & Sexuality.* 2011;13(6): 713-725.

Njue C, Voeten, H, Ahlberg BM. Youth in a void: Sexuality, HIV/AIDS and communication in Kenyan public schools. *Sex Education: Sexuality, Society and Learning.* 2011;11(4): 459-470.

Njue C, Voeten HA, Remes P. Disco funerals: a risk situation for HIV infection among youth in Kisumu, Kenya. *AIDS.* Feb 20 2009;23(4): 505-509.

Nobelius AM, Kalina B, Pool R, Whitworth J, Chesters J, Power R. Sexual and reproductive health information sources preferred by out-of-school adolescents in rural southwest Uganda. *Sex Education* 2010a;10(1): 91-107.

Nobelius AM, Kalina B, Pool R, Whitworth J, Chesters J, Power R. "You still need to give her a token of appreciation": the meaning of the exchange of money in the sexual relationships of out-of-school adolescents in rural southwest Uganda. *Journal of Sex Research.* Sep 2010b;47(5): 490-503.

Nobelius AM, Kalina B, Pool R, Whitworth J, Chesters J, Power R. Sexual partner types and related sexual health risk among out-of-school adolescents in rural south-west Uganda. *AIDS Care.* Feb 2011;23(2): 252-259.

Nobelius AM, Kalina B, Pool R, Whitworth J, Chesters J, & Power R. "The young ones are the condom generation": condom use amongst out-of-school adolescents in rural southwest Uganda. *Journal of Sex Research.* 2012;49(1):88-102.

Nzioka C. Unwanted pregnancy and sexually transmitted infection among young women in rural Kenya. *Culture, Health & Sexuality.* Jan 2004;6(1): 31-44.

Pfeiffer C, Kleeb M, Mbelwa A, Ahorlu C. The use of social media among adolescents in Dar es Salaam and Mtwara, Tanzania. *Reproductive Health Matters.* May 2014;22(43): 178-186.

Puffer ES, Watt MH, Sikkema KJ, Ogwang-Odhiambo RA, Broverman SA. The protective role of religious coping in adolescents' responses to poverty and sexual decision-making in rural Kenya. *Journal of Research on Adolescence : the Official Journal of the Society for Research on Adolescence.* Mar 1 2012;22(1): 1-7.

Sekiwunga R, Whyte SR. Poor parenting: teenagers' views on adolescent pregnancies in eastern Uganda. *African Journal Of Reproductive Health.* Dec 2009;13(4): 113-127.

Sommer M. Ideologies of sexuality, menstruation and risk: girls' experiences of puberty and schooling in northern Tanzania. *Culture Health & Sexuality.* 2009;11(4): 383-398.

Tolley EE, Kaaya S, Kaale A, et al. Comparing patterns of sexual risk among adolescent and young women in a mixed-method study in Tanzania: implications for adolescent participation in HIV prevention trials. *Journal of the International AIDS Society.* 2014;17(3 Suppl 2):19149.

Wagman J, Baumgartner JN, Waszak Geary C, et al. Experiences of sexual coercion among adolescent women: qualitative findings from Rakai district, Uganda. *Journal Of Interpersonal Violence.* Dec 2009;24(12): 2073-2095.
